# Neck and mind: exploring emotion processing in cervical dystonia

**DOI:** 10.3389/fnins.2025.1599951

**Published:** 2025-05-27

**Authors:** Federico Carbone, Marina Peball, Philipp Ellmerer, Beatrice Heim, Wolfgang Nachbauer, Elisabetta Indelicato, Matthias Amprosi, Philipp Mahlknecht, Anna Hussl, Anna Hotter, Roberta Granata, Klaus Seppi, Atbin Djamshidian, Sylvia Boesch

**Affiliations:** ^1^Department of Neurology, Medical University Innsbruck, Innsbruck, Austria; ^2^Department of Neurology, District Hospital Kufstein, Kufstein, Austria

**Keywords:** cervical dystonia, eye-tracking technology, facial recognition, emotions, neuropsychological test

## Abstract

**Objective:**

A wide range of non-motor symptoms such as pain, mood disorders, insomnia, and executive dysfunction may occur in focal dystonia. Little is known, however, about emotional processing. We aim to assess emotion recognition and alexithymia in patients with cervical dystonia (CD) compared to healthy age-, sex- and education-matched controls (HC).

**Methods:**

Emotion processing was assessed with an eye-tracking paradigm using a validated dataset of facial expressions and the Toronto Alexithymia Scale (TAS-20). Dystonia severity and disability, cognition, and comorbid depression and anxiety were also assessed.

**Results:**

We recruited 35 CD patients and 17 matched HC. In the eye-tracking task, CD patients recognized emotions less accurately than HCs (77.0% vs. 84.4%; *p* = 0.001), primarily based on difficulties in identification of fear (*p* = 0.003) and surprise (*p* = 0.037). Moreover, patients had longer fixations within the mouth region (*p* = 0.027) and left eye (*p* = 0.037) than HC. CD patients also had significantly higher total TAS-20 scores (*p* = 0.002) and subscores (difficulty identifying and describing feelings; all *p* ≤ 0.026). Five patients (14.3%) reached the threshold for alexithymia and 6 (17.1%) for possible alexithymia. No HC scored positive for alexithymia and only 2 (11.8%) did for possible alexithymia. TAS-20 score correlated inversely with emotion recognition task performance (*r* = −0.411; *p* = 0.014).

**Interpretation:**

We found poorer performance in emotion recognition in CD patients compared to HC. Together with a different gaze pattern and higher scores for alexithymia our results highlight deficits in emotion processing in CD.

## 1 Introduction

Isolated Cervical Dystonia (CD) is the most common adult-onset form of dystonia and is characterized by abnormal head and neck posturing caused by involuntary muscle contractions (Albanese et al., 2023). Dystonia was traditionally seen as a basal ganglia disorder. However, recent neuroimaging and-physiology studies reveal that CD may be caused by a dysfunctional complex network involving the brain stem, cortex, cerebellum, and basal ganglia (Albanese et al., 2023; O'Connor et al., 2024). Therefore, it is not surprising that CD patients can also suffer from a variety of non-motor complaints such as depression, pain, anxiety, or sleep disturbances, which often result in reduced quality of life (QoL; Albanese et al., 2023; O'Connor et al., 2024). Executive dysfunction and aspects of social cognition (Czekoova et al., 2017) have been studied in CD but less is known about other cognitive domains (O'Connor et al., 2024; Defazio et al., 2024). Particularly, studies focusing on emotion recognition are scarce (Coenen et al., 2024). Emotional processing combines the ability to percept social information (e.g., facial expressions), to relate feelings or intentions to behavior of oneself or others (e.g., theory of mind), and to be empathetic. It is the cornerstone of understanding and adapting behavior in social contexts (Coenen et al., 2024; Adolphs, 2001). The inability to recognize other people’s emotions from their facial expressions is related to alexithymia. Alexithymia describes a personality trait characterized by difficulties recognizing, differentiating and describing emotional states, an externally-orientated thinking style, and limited imaginative capacity. Alexithymia is common in psychiatric and neuropsychological disorders (Coenen et al., 2024; Pisani et al., 2021). Data on the prevalence and impact in neurological disorders mostly focused on patients with Parkinson’s Disease (Ricciardi et al., 2015).

The presence of alexithymia may complicate emotional processing and regulation in CD patients, potentially diminishing coping mechanisms and increasing psychological distress. Consequently, it may hinder interpersonal relationships, contribute to social withdrawal, and exacerbate psychiatric comorbidities such as anxiety disorders or depression, which are already prevalent in this population. Studying emotion recognition in CD is therefore essential for understanding the full scope of cognitive and social impairments these patients face that mostly are in the prime of their life during disease evolution. We believe that impaired emotional processing in CD is an integral part of the neurodegenerative process impairing the mentioned neuronal network, not merely a secondary consequence of motor symptoms.

Hence, in this study, we aimed to assess emotional recognition and alexithymia in patients with CD compared to age-, sex-, and education-matched healthy controls (HC). For assessment, we used the 20-item Toronto Alexithymia Scale (TAS-20; Bagby et al., 1994) as a self-reported questionnaire. In people with alexithymia, answering questions on emotions and reproducing emotional states characteristically is difficult (Ricciardi et al., 2015). To overcome these challenges, we added an eye-tracking task to assess recognition of facially expressed emotions as an objective measurement. Based on previous studies in CD, we hypothesize that emotion recognition is impaired in CD patients, independent of dystonia severity or emotional state. By highlighting the link between CD and dysfunctional emotion recognition, this research underscores the need for a more holistic, interdisciplinary approach to CD management and contributes to a broader understanding of the disorder’s multifaceted nature.

## 2 Methods

### 2.1 Participants

In this single-center pilot study, we consecutively included patients with isolated idiopathic adult-onset CD (Albanese et al., 2023) from our specialized outpatient department as well as age-, sex-, and education-matched HCs. Other exclusion criteria were selected for possible bias on the outcome measurements and included a Mini–Mental State Examination (MMSE; Folstein et al., 1975) score below 26 points, psychiatric disorders, and an uncorrected visual impairment. Participants were not allowed to have drugs affecting the central nervous system, except for antidepressants if on a stable regimen for at least 4 weeks prior to testing.

The study was approved by the local ethics committee (approval number: AN1979 336/4.19401/5.10 [4464a]). All individuals gave written informed consent before participation. No subject received a stipend for participation. All procedures were performed in accordance with the 1964 Helsinki declaration and its later amendments.

### 2.2 Clinical assessment

Demographic data of all participants was taken by interview and included sex, age, smoking status, and education years. CD patients were evaluated for disease duration, the presence of tremor, type of dystonia (phasic vs. tonic), and receiving botulinum neurotoxin (BoNT) therapy. Clinical assessment of CD patients was performed using a modified version of the Tsui scale (Poewe et al., 1998; Tsui et al., 1986) and the Toronto Western Spasmodic Torticollis Rating Scale (TWSTRS; Boyce et al., 2012). The subscales of the TWSTRS for severity, disability, and pain were also calculated.

The Hospital Anxiety and Depression Scale (HADS; maximum score of 21 points for the subscales for depression and anxiety; higher points represent worse symptom burden; Zigmond and Snaith, 1983) was used. A cut-off value of ≥8 points in the HADS anxiety (−A) or depression (-D) subscale suggests the presence of clinical anxiety disorders or depression (Zigmond and Snaith, 1983). To examine alexithymia, the TAS-20 (Bagby et al., 1994) was administered in all participants. A score of 52 to 60 points defines “possible alexithymia,” while a score of 61 to 100 points is positive for “alexithymia.” The TAS-20 has three subscales: factor I describing a difficulty identifying feelings (test items: 1, 3, 6, 7, 9, 13, 14), factor II regarding a difficulty describing feelings (test items: 2, 4, 11, 12, 17), and factor III for externally-oriented thinking (test items: 5, 8, 10, 15, 16, 18, 19, 20). The TAS-20 is the most frequently used instrument for alexithymia assessment in clinical and non-clinical settings with satisfactory convergent and discriminant validity as well as high agreement to observer ratings (Bagby et al., 1994).

### 2.3 Eye-tracking assessment

The facial emotion recognition task was performed in all subjects using the Tobii TX-300 eye tracker with Tobii Pro Studio Software 3.4.8.1348. All participants were tested by the same investigator with identical light conditions. Examination was performed in a windowless and quiet room to minimize distractions. The task was presented on a 23″ screen at a measured viewing distance of 64 cm. Eye positions on the screen were recorded by the eye-tracker as raw data which were calculated by a software algorithm to determine saccades and fixations. Saccades are movements of the eyes from one point to another and fixation describes a halt in eye movement to obtain visual information. In this study, a temporal cut-off of 50 ms was used to distinguish between saccades (<50 ms) and fixations (>50 ms; Negi and Mitra, 2020). Before performing the emotion recognition task, the eye-tracker was individually calibrated for every subjects. Before the display of any emotion, patients had to fixate on a decentralized cross for 2 s to avoid a systematic bias for central exploitation. All 42 facial emotions were displayed for the same length of 4,000 ms in the same order for every participant. Displayed emotions could be neutral, disgust, joy, anger, surprise, fear, or despair (sadness). The displayed faces were derived from the NimStim set of facial expressions, a validated set used for emotion recognition studies (Tottenham et al., 2009). After the display, a screen with all possible answers was shown from which subjects had to choose their correct answer and verbally communicate it to the investigator. The % of correctly identified emotions was calculated. Besides the correct answer in %, the time to first fixation on an area of interest (face, left eye, right eye, mouth, nose; in sec), the total fixation duration on the respective area (in sec), the average duration of one fixation on the respective area (in sec), and the fixation count (overall) were calculated. The face region included all areas not specified under the other categories. If the time to first fixation on an area of interest was <0.1 s, the test was excluded, as this implied noncompliance to fixate on the decentralized cross before exploring the face.

Eye-tracking offers a valuable, objective complement to traditional paper-based questionnaires in examining alexithymia. While questionnaires like the TAS-20 rely on introspection and subjective awareness—which are inherently impaired in alexithymia (Ricciardi et al., 2015)—eye-tracking allows direct observation of how individuals visually attend to emotional stimuli (e.g., facial expressions). This provides insight into unconscious or automatic processing of emotional information, revealing attentional biases or avoidance patterns that are not captured through self-report. In this study with CD patients, eye-tracking can help disentangle whether deficits in emotion recognition stem from perceptual inattention, cognitive processing issues, or emotional detachment. Thus, integrating eye-tracking enhances the diagnostic validity of alexithymia assessment which justifies the approach used in this study.

### 2.4 Statistical analyses

Categorical variables are given in number (n) and percent (%) of the category (sex, smoking status, alexithymia, correct answer on the eye-tracking task, CD origin, presence of tremor, BoNT therapy). For continuous quantitative measures the mean with its standard deviation were calculated. Group comparisons were made between CD patients and HCs. For categorical data, group comparisons were based on Qui-square test. Continuous variables were tested for normality with the Kolmogorov–Smirnov test and Mann–Whitney-U test or unpaired student’s t-test were used for group comparisons depending on the scale type (see Table legends for details). Spearman’s rank-order correlation (r) was used to test for any relationship of total TAS-20 score (not normally distributed) with the total result of the eye-tracking emotion recognition task as well as TAS-20 with CD severity (TWSTRS and subscores, Tsui Score), MMSE, emotional state (HADS-A, HADS-D), and disease duration. Correlation coefficient (r) absolute values between 0 and 0.09 are a negligible correlation, 0.1 to 0.39 a weak correlation, 0.4 to 0.69 a moderate correlation, 0.7 to 0.89 a strong correlation, and 0.9 to 1.0 a very strong correlation (Schober et al., 2018). The significance level was set at a two-sided *p*-value of <0.05. Due to the exploratory nature, a correction for multiple testing was not applied. If adjustments would be made for multiple comparisons, the set *p*-values according to Bonferroni’s correction are displayed in the table legends. SPSS 25.0 for windows (SPSS Inc., IBM Corporation and other(s) 1989, 2013, Chicago, IL) was used to analyze data.

## 3 Results

We included 35 patients with isolated CD and 17 age-, sex-, and education-matched HCs. Demographic characteristics are summarized in Table 1. All patients received treatment with BoNT. The mean time since last BoNT injection was 101.3 ± 12.2 days. CD patients had higher overall HADS-A and HADS-D subscores (6.2 ± 3.4 points and 4.5 ± 3.3 points) compared to HC subjects (3.4 ± 2.3 points and 1.7 ± 1.8 points; *p* = 0.001 and *p* = 0.002 respectively). Using predefined cut-off values, 9 (25.7%) CD patients had a HADS-A score suggestive of clinical anxiety disorder and 6 (17.1%) CD patients had a HADS-D result suggestive of clinical depression. Only 1 HC scored positive for anxiety disorders and none for depression.

**Table 1:** Demographic and clinical data of the study population.

	**CD patients (*n*=35)**	**Controls (*n*=17)**	***p*-value**
Female sex, n (%)	26 (74.3)	11 (64.7)	0.525
Current smoker, n (%)	24 (68.6)	9 (52.9)	0.272
Age (years)	60.8 ± 9.7	55.7 ± 11.4	0.122
MMSE	29. 0± 1.1	29.1 ± 0.9	0.622
Education (years)	11.7 ± 2.6	11.7 ± 1.8	0.877
HADS-A	6.2 ± 3.4	3.4 ± 2.3	0.001
≥ 8 points, n (%)	9 (25.7)	1 (5.9)	0.089
HADS-D	4.5 ± 3.3	1.7 ± 1.8	0.002
≥ 8 points, n (%)	6 (17.1)	0 (0)	0.070
TAS-20 Total Score	48.2 ± 10.9	38.5 ± 8.7	0.002
TAS-20 Factor I	16.0 ± 6.1	10.1 ± 3.4	<0.001
TAS-20 Factor II	11.7 ± 3.2	9.4 ± 3.4	0.026
TAS-20 Factor III	20.5 ± 4.9	19.1 ± 4.0	0.260
Alexithymia per TAS-20, n (%)	5 (14.3)	0 (0)	0.199
Possible alexithymia per TAS-20, n (%)	6 (17.1)	2 (11.8)	
No alexithymia per TAS-20, n (%)	25 (68.6)	15 (88.2)	
CD Origin - Idiopathic, n (%)	35 (100.0)	NA	NA
Disease duration (years)	16.7 ± 8.7	NA	NA
Tremor present, n (%)	11 (31.4)	NA	NA
Botulinum neurotoxin therapy, n (%)	33 (94.3)	NA	NA
Time since last botulinum toxin injection (days)	101.3 ± 12.2	NA	NA
Tsui Total Score	4.9± 3.0	NA	NA
TWSTRS Total Score	29.1 ± 8.8	NA	NA
TWSTRS Severity Scale	15.1 ± 4.8	NA	NA
TWSTRS Disability Scale	6.8 ± 3.6	NA	NA
TWSTRS Pain Scale	10.3 ± 8.2	NA	NA

Continuous data are presented as mean ± standard deviation; categorical data as n, %. Group comparisons with unpaired t-test or Mann–Whitney-U test for continuous data (all data normally distributed except for TAS-20 Total Score and TAS-20 Factor I) and Qui-square test for categorical data. Significance level: two-sided *p* ≤ 0.05. If adjusting for multiple comparisons, the *p*-values would be set at *p* < 0.00625 (0.05/8, not considering the subscores of the TAS-20). CD, cervical dystonia; MMSE, Mini Mental State Examination; HADS-A/D, Hospital Anxiety And Depression Scale – Anxiety/Depression Subscale; TAS-20; 20-item Toronto Alexithymia Scale; TWSTRS, Toronto Western Spasmodic Torticollis Rating Scale; NA, not applicable. TAS-20 Factor I: Difficulty identifying feelings (test items 1, 3, 6, 7, 9, 13, 14); TAS-20 Factor II: Difficulty describing feelings (test items 2, 4, 11, 12, 17); TAS-20 Factor III: Externally-oriented thinking (test items 5, 8, 10, 15, 16, 18, 19, 20).

Patients with CD had a significantly higher mean TAS-20 Total Score (48.2 ± 10.9 points) compared to HCs (38.5 ± 8.7 points; *p* = 0.002). TAS-20 factor I, assessing difficulty in identifying feelings, was also higher in CD patients (16.0 ± 6.1 points) vs. HC subjects (10.1 ± 3.4 points; *p* < 0.001), as was TAS-20 factor II, i.e., difficulty describing feelings (11.7 ± 3.2 points vs. 9.4 ± 3.4 points; *p* = 0.026). There was no statistically significant difference in the score of factor III of the TAS-20, i.e., externally-oriented thinking (*p* = 0.260). Five CD patients (14.3%) reached the threshold for alexithymia (61–100 points) and six patients (17.1%) did for possible alexithymia (52–60 points). No HC scored positive for alexithymia and only 2 (11.8%) did for possible alexithymia (*p* = 0.199). HADS-A and HAD-D scores also differed significantly between groups (*p* < 0.002; Table 1). Total TAS-20 score significantly correlated with overall HADS-A (*r* = 0.497; *p* < 0.001) and HADS-D (*r* = 0.677; *p* < 0.001) as well as dichotomized (cut-off ≥8 points) HADS-A (*r* = 0.108; *p* = 0.003) and HADS-D (*r* = 0.359; *p* = 0.009), but not with the TWSTRS Total score (*r* = 0.123; *p* = 0.482), TWSTRS Severity subscore (*r* = 0.096; *p* = 0.582), TWSTRS Disability subscore (*r* = 0.075; *p* = 0.670), TWSTRS Pain subscore (*r* = 0.171; *p* = 0.326), Tsui score (*r* = 0.123; *p* = 0.482), MMSE (*r* = 0.077; *p* = 0.590), or disease duration (*r* = −0.222; *p* = 0.201). TAS-20 score also correlated inversely with emotion recognition (*r* = −0.411; *p* = 0.014; Table 2). In the eye-tracking task, CD patients identified significantly less emotions correctly than HCs (77.0 ± 8.5% vs. 84.4 ± 6.2%; *p* = 0.001). CD patients recognized surprise (87.2 ± 15.5% vs. 94.2 ± 8.2%; *p* = 0.037) and fear (42.5 ± 22.6% vs. 60.2 ± 17.7%, *p* = 0.003) significantly less than HC. There was no significant difference in recognizing disgust (*p* = 0.082), joy (*p* = 0.960), neutral faces (*p* = 0.142), anger (*p* = 0.070), and despair (*p* = 0.459). We found differences in facial exploration patterns. CD patients had longer fixations within the mouth region (*p* = 0.027) and the left eye (*p* = 0.037) compared to that of HCs. There was, however, no difference in the average duration of one fixation on the whole face (*p* = 0.765), right eye (*p* = 0.874), and nose (*p* = 0.709) between patients and HCs. Moreover, there was no statistically significant difference in total fixation duration of any area of interest (face, left eye, right eye, mouth, nose; all *p* > 0.323), time to first fixation of any region (all *p* > 0.085) and fixation count (all *p* > 0.128) between the groups (Table 2). Correlations analyses revealed no differences between HADS-A, HADS-D and any eye tracking tasks (all *p* > 0.1).

**Table 2:** Results of the eye-tracking emotion recognition task and facial exploration strategies.

Rate % of correct emotion recognition		CD patients (*n* = 35)	Controls (*n* = 17)	*p*-value
Disgust		80.1 ± 20.1	89.2 ± 15.5	0.082
Joy		96.2 ± 8.2	96.1 ± 7.2	0.960
Neutral		94.9 ± 14.1	99.1 ± 4.0	0.142
Anger		89.1 ± 17.4	96.1 ± 9.8	0.070
Surprise		87.2 ± 15.5	94.2 ± 8.2	0.037
Fear		42.5 ± 22.6	60.2 ± 17.7	0.003
Despair		53.4 ± 24.9	58.9 ± 24.4	0.459
Total		77.0 ± 8.5	84.4 ± 6.2	0.001
Facial exploration strategy	AOI			
Total fixation duration (in sec)	Face	3.16 ± 0.60	3.23 ± 0.40	0.650
	Left Eye	0.72 ± 0.41	0.77 ± 0.59	0.717
	Right Eye	0.66 ± 0.44	0.74 ± 0.44	0.536
	Mouth	0.80 ± 0.58	0.66 ± 0.36	0.365
	Nose	0.80 ± 0.68	0.99 ± 0.56	0.323
Time to first fixation (in sec)	Face	0.16 ± 0.09	0.15 ± 0.10	0.795
	Left Eye	1.40 ± 0.79	1.00 ± 0.61	0.085
	Right Eye	1.12 ± 0.61	1.06 ± 0.50	0.721
	Mouth	1.18 ± 1.01	0.98 ± 0.42	0.462
	Nose	0.76 ± 0.62	0.58 ± 0.29	0.238
Average fixation duration of the respective AOI (in sec)	Face	0.34 ± 0.11	0.33 ± 0.12	0.765
	Left Eye	0.34 ± 0.09	0.32 ± 0.17	0.037
	Right Eye	0.32 ± 0.08	0.32 ± 0.15	0.874
	Mouth	0.37 ± 0.17	0.29 ± 0.06	0.027
	Nose	0.29 ± 0.12	0.30 ± 0.27	0.709
Fixation count (overall)	Face	9.88 ± 2.09	10.78 ±2.08	0.152
	Eyes	2.10 ± 1.11	2.36 ± 0.97	0.412
	Mouth	2.14 ± 1.10	2.31 ± 1.37	0.629
	Nose	2.59 ± 1.55	3.24 ± 1.17	0.128

Emotion recognition represent % of correct identification of facial expression associated with the respective emotion. All data are presented as mean ± standard deviation. Group comparisons with unpaired t-test or Mann–Whitney-U test for continuous data (all normally distributed except for total duration of fixation on the left eye). Significance level: two-sided *p* ≤ 0.05. If adjusting for multiple comparisons, the *p*-values would be set at *p* < 0.007 (0.05/7) for the emotion recognition task and *p* < 0.01 (0.05/5) or *p* < 0.0125 (0.5/4) for the facial exploration strategies. CD, cervical dystonia; AOI, area of interest; n, number. The face region includes all other areas of interest not specified.

## 4 Discussion

We found significantly worse emotional recognition in patients with CD compared to HCs. More specifically, alexithymia was significantly more common in CD. The subdomain assessment showed significantly more difficulties in CD patients with identifying and describing feelings.

Furthermore, CD patients recognized emotions less accurately than HC, mostly based on impairments in surprise and fear identification, which was independent of disease duration. Results of the eye-tracking analysis and TAS-20 showed a moderate inverse correlation, i.e., meaning that higher alexithymia scores correlated with a lower correct identification of facial expressions on the eye-tracker. Facial exploration also revealed that CD patients had a longer fixation on the mouth and left eye regions but there were no other differences to HCs.

These findings have several important clinical implications in CD. The higher prevalence of alexithymia and significantly poorer emotion recognition highlight the need for routine screening of emotional and cognitive functioning in CD patients. These deficits appear to not merely be byproducts of motor symptoms but to represent core features that can substantially impact (psychosocial) well-being and interpersonal relationships. The observed impairments in recognizing specific emotions such as fear and surprise, which are crucial for social communication and threat detection, suggest that CD patients may struggle in real-life social interactions, further contributing to social withdrawal and reduced QoL.

The moderate inverse correlation between TAS-20 scores and emotion recognition accuracy on the eye-tracking task supports the validity of using multimodal assessment tools in clinical settings. Incorporating eye-tracking as a diagnostic tool may help with detecting emotional processing deficits that may otherwise be unrecognized in self-report measures alone, especially in individuals with high alexithymic traits. In our study, the absence of a central fixation bias strengthens the interpretation of the eye-tracking findings. It indicates that CD patients focus their gaze on regions with potential key information (mouth, eyes) rather than the center of the visual stimulus. This suggests intact intentional attention to specific emotional cues and thus defect emotional processing itself rather than default visual behavior. Taken together, these results advocate for a more comprehensive, interdisciplinary approach to CD treatment, integrating neurologic, psychological, and social components. Tailored interventions, such as emotion recognition training or psychotherapeutic support addressing alexithymia, may enhance coping strategies and ultimately improve patient outcomes beyond the motor domain.

Previous studies assessing facial emotion recognition in CD patients yielded inconsistent results with most showing impairment (Rinnerthaler et al., 2006; Ellement et al., 2021; Burke et al., 2020; Mahady et al., 2023) and others normal function (Coenen et al., 2024). These discrepancies may be explained by methodological differences, e.g., in assessment methods or duration of stimuli presentation [e.g., computer-based tests vs. questionnaires; NimStim set of facial expressions, stimulus presentation for 4,000 ms in our study vs. computer-based Facially Expressed Emotion Labeling test; stimulus presentation for 300 msec in Rinnerthaler et al. (2006)], small sample sizes, or differences in patient characteristics. For example, in our study the disease duration as well as the severity were significantly higher than in another study where using the computer-based Facial Expression of Emotion: Stimuli and Tests revealed normal emotion processing (16.7 ± 6.1 vs. 10.84 ± 8.15 years; Coenen et al., 2024). Our results are in line with one previous study, which also showed deficits in recognizing fearful facial expressions using the Affect Naming Task from the Advanced Clinical Solutions for the Wechsler Adult Intelligence Scale IV. They also showed difficulties in identifying sadness, anger, and surprise compared to happiness and disgust (Ellement et al., 2021). However, and in contrast to this study, eye tracking was not assessed. CD disease duration was also not provided. Other trials studying overall emotion recognition found that CD patients experience difficulties in naming facial expressions [computer-administered Name Face Affect (picture) task of the Florida Affect Battery and Cambridge Mindreading Face-Voice Battery] compared to HCs (Burke et al., 2020; Mahady et al., 2023) and to normative data (Monaghan et al., 2021). Identification of facial expressions was difficult for almost 25% of the CD patient cohort in the second study (Monaghan et al., 2021), which is comparable to the error rate found in our trial.

Our findings support the concept of CD being a network disorder manifesting with motor as well as non-motor symptoms. Involvement of the corpus callosum, thalamic projections, inferior fronto-occipital fasciculus, and inferior temporal and superior orbital gyri were found in CD patients, all of which are important for processing interhemispheric information, sensorimotor information, and executive control (Valeriani and Simonyan, 2020). In alexithymia, which involves processing of emotional sensory input, a dysfunction of the collicular-pulvinar-amygdala subcortical pathway was proposed as pathological mechanism in CD patients (Hutchinson et al., 2018). This pathway is believed to mediate non-conscious perception of affective stimuli such as emotions, especially negative stimuli of different sensory modalities, resulting in emotional processing and consecutive actions via multiple neural networks (Kragel et al., 2021; Pessoa and Adolphs, 2010).

Depression and anxiety are important comorbidities in CD patients (O'Connor et al., 2024) and linked to alexithymia (Preece et al., 2024). This is supported in our findings and results from other studies assessing facial emotion recognition (Mahady et al., 2023). Alexithymia represents a reduced capacity to accurately identify an emotion. The correct identification of an emotion is believed to be crucial to activate and facilitate down-stream emotion regulation decisions (e.g., which emotion strategy to use as response to an input; Preece et al., 2024). Not only may difficulties during this process lead to negative long-term emotional outcomes such as anxiety disorders or depression, but the psychopathological symptoms themselves may negatively affect or limit the person’s capability to correctly recognize emotional input (Preece et al., 2024). Diagnosing and treating anxiety disorders and depression in CD patients may thus not only improve their overall QoL (Smit et al., 2016), but also social cognition and thus participation, highlighting the clinical importance.

Alexithymia was independent of cognition (MMSE) and motor burden (TWSTRS, Tsui score) in our study, which also confirms previous results (Defazio et al., 2024; Coenen et al., 2024; Ellement et al., 2021; Burke et al., 2020; Monaghan et al., 2021).

Our study has several limitations. As this study was exploratory in nature, a power calculation was not performed. The small sample size may be subject to a sample bias and thus limit generalizability. However, the sample size of our study cohorts is comparable to previous studies on the subject. A larger sample size may be required for further studies to broaden the applicability of the results and to capture the heterogeneity present in the CD population, such as variations in symptom severity, comorbidities, or influential factors. Depressive symptoms, and disease-related pain and disability are known to be among the most important predictors of QoL in the CD population (Smit et al., 2016). However, difficulties in emotion recognition and thus social interaction are also likely to contribute to worsened QoL. Since we did not administer any measure on health-related QoL, no inferences can be made with regard to this relationship on the basis of our data. Important strengths must be noted. All CD patients were thoroughly assessed by movement disorder specialists and clinical ratings were compared to a HC group. The study also included assessment of depression and anxiety as important comorbidities. Moreover, to test for alexithymia and facial emotion recognition, we used not only a self-reported questionnaire, but also eye-tracking as an objective measure. Eye-tracking is non-invasive, time-and cost-effective and therefore has the potential to be a simple biomarker of disease in both research and a clinical setting.

## 5 Conclusion

We found a high prevalence of possible and definite alexithymia in CD (31%) compared to HCs (12%), especially regarding the identification and description of feelings. Additionally, recognition of facial emotional expressions was impaired in the eye-tracking analysis, mostly due to difficulties with recognition of surprise and fear. Visuospatial facial exploration appears to be intact in CD patients without a central fixation bias. This is the first study to use an eye-tracking task for objective assessment of emotion recognition and facial exploration strategies in CD patients. This study highlights that CD patients also have difficulties in social cognition. Previous studies have shown how CD patients may have issues in recognizing other’s emotions and intentions (i.e., impairments in the Theory of Mind). Our results show that these patients may also have similar issues in the perception of their own emotions (alexithymia). This lack of affective perception, coupled with difficulties with correct identification of facial emotional expressions may impair social interaction and participation and thus the quality of life of the affected patients.

Larger and more diverse studies are needed to demonstrate the general applicability of this findings in CD patients, to examine alexithymia in the context of QoL and social withdrawal, to solidify the added value of eye-tracking for identification of alexithymia in clinical settings, and to assess targeted treatment options.

## Data Availability

The raw data supporting the conclusions of this article will be made available by the authors upon reasonable request, without undue reservation.
